# LncRNAs expression signatures of cadmium-induced malignant transformation of human bronchial epithelial cells revealed by microarray

**DOI:** 10.1186/1756-8935-6-S1-P90

**Published:** 2013-03-18

**Authors:** Zhi-heng Zhou, Cai-xia Wang, Yi-xiong Lei, Hai-bai Liu, Min Wang

**Affiliations:** 1 School of public health, Guangzhou Medical University, Guangzhou 510182, People’s Republic of China; 2 Department of Internal Medicine of the First Hospital of Guangzhou, Guangzhou 510180, China

## Background

Cadmium (Cd) and its compounds are well-known environmental carcinogens, but the mechanisms underlying the carcinogenesis are not entirely understood yet. Long noncoding RNAs (IncRNAs) are an important class of pervasive genes involved in a variety of biological functions. They are aberrantly expressed in many types of cancers. In this study, we described IncRNAs profiles in 35^th^ passage of CdCI_2_ malignant transformation cells(35^th^ cell) and untransformed human bronchial epithelial cells(16HBE) by microarray.

## Methodology/principal findings

With abundant and varied probes accounting 33,045 LncRNAs in our microarray, the number of IncRNAs that expressed at a certain level could be detected is 21409. From the data we found there were 369 IncRNAs were upregulated and 90 IncRNAs were downregulated (≥2.0fold-change, P<0.05) in 35^th^ cells compared with 16HBE cells. Our data showed that upregulated IncRNAs were more common than downregulated ones. ENST00000477387, ENST00000394732, ENST00000485873, ENST00000497538, uc002odz.1, AK023660, NR_023938, BC019085 were evaluated by qPCR in 35th cells compared with 16HBE cells. The eigth IncRNAs were aberrantly expressed in 35th cells compared with matched 16HBE cells.

## Conclusions/significance

Our study is the first one to determine genome-wide IncRNAs expression patterns in Cadmium-induced malignant transformation by microarray. The results displayed that clusters of IncRNAs were aberrantly expressed in CdCI_2_ malignant transformation cells compared with 16HBE cells, which revealed that IncRNAs differentially expressed in CdCI_2_ malignant transformation cells may exert a partial or key role in cadmium-induced cancers. Taken together, this study may provide a viable mechanism for cadmium-induced cancers.

